# Supplementary material for: Personalized recurrence risk assessment following the birth of a child with a pathogenic de novo mutation
Source: Nat Commun. 2023 Feb 15;14:853. doi: 10.1038/s41467-023-36606-w (PMC9932158; doi:10.1038/s41467-023-36606-w)
Supplement: Supplementary file 1 — Supplementary Information [file 41467_2023_36606_MOESM1_ESM.pdf]

**Supplementary Material**

# Personalized recurrence risk assessment following the birth of a child with a *de novo* pathogenic mutation

Bernkopf, Abdullah *et al.*

## Content:

Page 2: Supplementary Fig. S1: The timing of occurrence of the DNM impacts on recurrence risk.

Page 4: Supplementary Fig. S2: Deep-sequencing results for all biological samples analyzed in the mosaic families.

Page 5: Supplementary Fig. S3: Downsampled Deep-NGS results for selected biological samples in families FAM27 (A-B) and FAM34 (C-D).

Page 6: Supplementary Note 1: Stratification of DNMs into 7 categories

Page 10: Supplementary Note 2: Design and results for assessment of mosaicism in two families with the larger indel DNMs (FAM12b and FAM54)

Page 12: Supplementary Note 3: Sensitivity of the Deep-NGS assay for low VAF quantification

Page 14: Supplementary Note 4: Successes and failures in resolving haplotypes using long-read sequencing (MinION platform from ONT)

Page 16: Supplementary Note 5: Allele-specific PCR for haplotyping the DNM in *AHDC1* in FAM38

Page 17: Supplementary Note 6: Estimating the recurrence risk associated with mosaicism from multi-sibling families and sperm WGS data

Page 22: Supplementary Note 7: Choice of biological samples to analyze for mosaicism

Page 23: Supplementary References

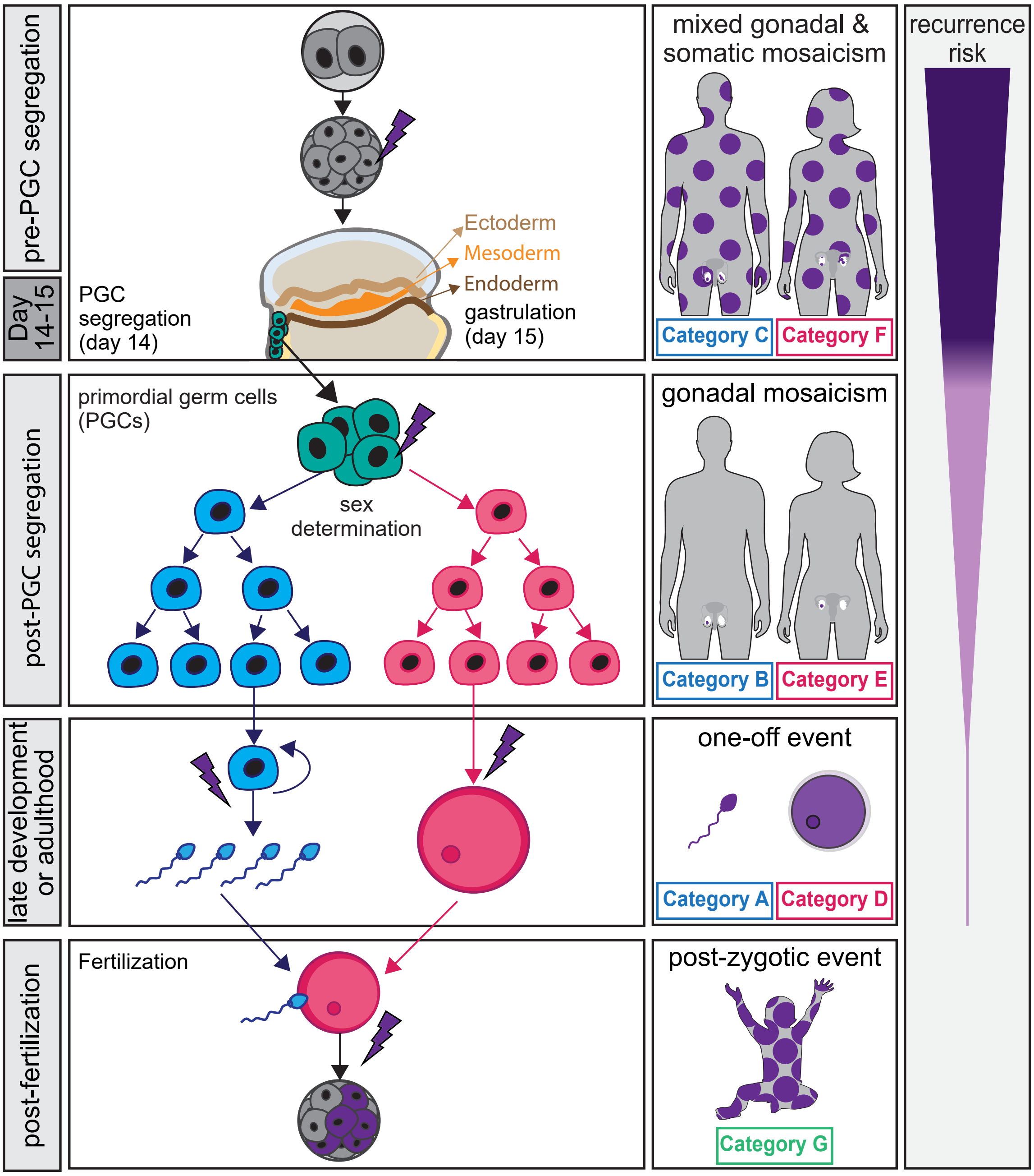


**Supplementary Fig. S1: The timing of occurrence of the DNM impacts on recurrence risk.** De novo mutations (DNMs) can occur at any point prior to, or during the development of, the embryo, potentially resulting in mosaicism. If the DNM arises in a parent prior to the segregation of primordial germ cells (PGCs) and gastrulation (days 14-15 of embryogenesis), it can result in mixed mosaicism affecting both the somatic tissues and gonads in either the father (Category C in this study) or the mother (Category F); because they occur very early, these scenarios are associated with the highest sibling recurrence risk. If the DNM arises in parental PGCs after PGC-soma segregation, mosaicism will be confined to the germline lineage in the father (Category B) or the mother (Category E); these circumstances are associated with intermediate sibling recurrence risks, depending on the relative proportion of mutant cells. Because these mosaic events occur before sex determination, they affect both sexes equally. If the DNM arises as a one-off mutational event in late development or adulthood in the father (Category A) or the mother (Category D), the recurrence risk is negligible. The DNM may also arise as a post-zygotic event in the child following fertilization (Category G); in this case, the recurrence risk is zero, as the DNM was never present in the parental

gametes. It is possible to quantify the recurrence risk for DNMs in Categories A-C and G, but the recurrence risk cannot be quantified for DNMs of maternal origin (Categories D-F) as oocytes cannot be accessed without invasive sample collection.

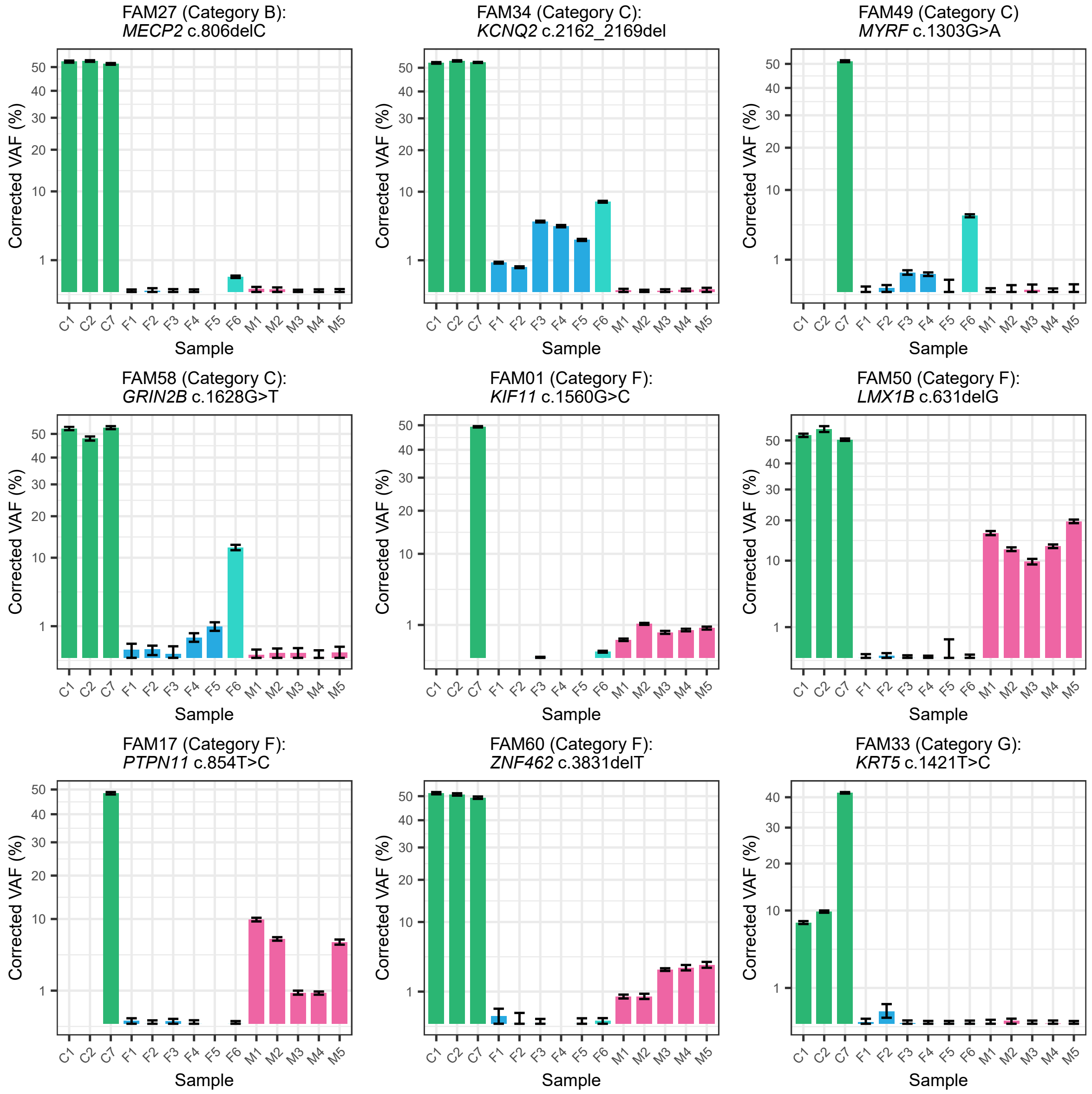


**Supplementary Fig. S2: Deep-sequencing results for all biological samples analyzed in the mosaic families**. Variant allele frequencies (VAF) measured for each tissue sample analyzed from the three family members and three unrelated controls plotted as the square root (sqrt). Family number, category, affected gene and coordinates of the DNM are indicated above each plot. The origin of the different tissue samples is indicated on the x-axis and colors reflect the parental/post-zygotic origin of the DNM [blue (somatic paternal tissues), turquoise (paternal sperm), pink (somatic maternal tissues), green (post-zygotic/proband tissues), gray (control)]. The ╳ represents a sample failure. Each bar represents a single VAF calculated from the sum of 3 technical replicates, then corrected using measurements from 3 unrelated controls (corrected VAFs, see Methods). Error bars represent the 95% binomial confidence intervals. Full data and controls are presented in Supplementary Data 2. FAM17 and FAM60 are the two families with multiple affected pregnancies and belong to category F (maternal mixed mosaicism). Abbreviations: F=father; M=mother; C=child; 1=buccal swab (left); 2=buccal swab (right); 3=blood; 4=saliva; 5=urine; 6=sperm; 7=genomic DNA from original testing.

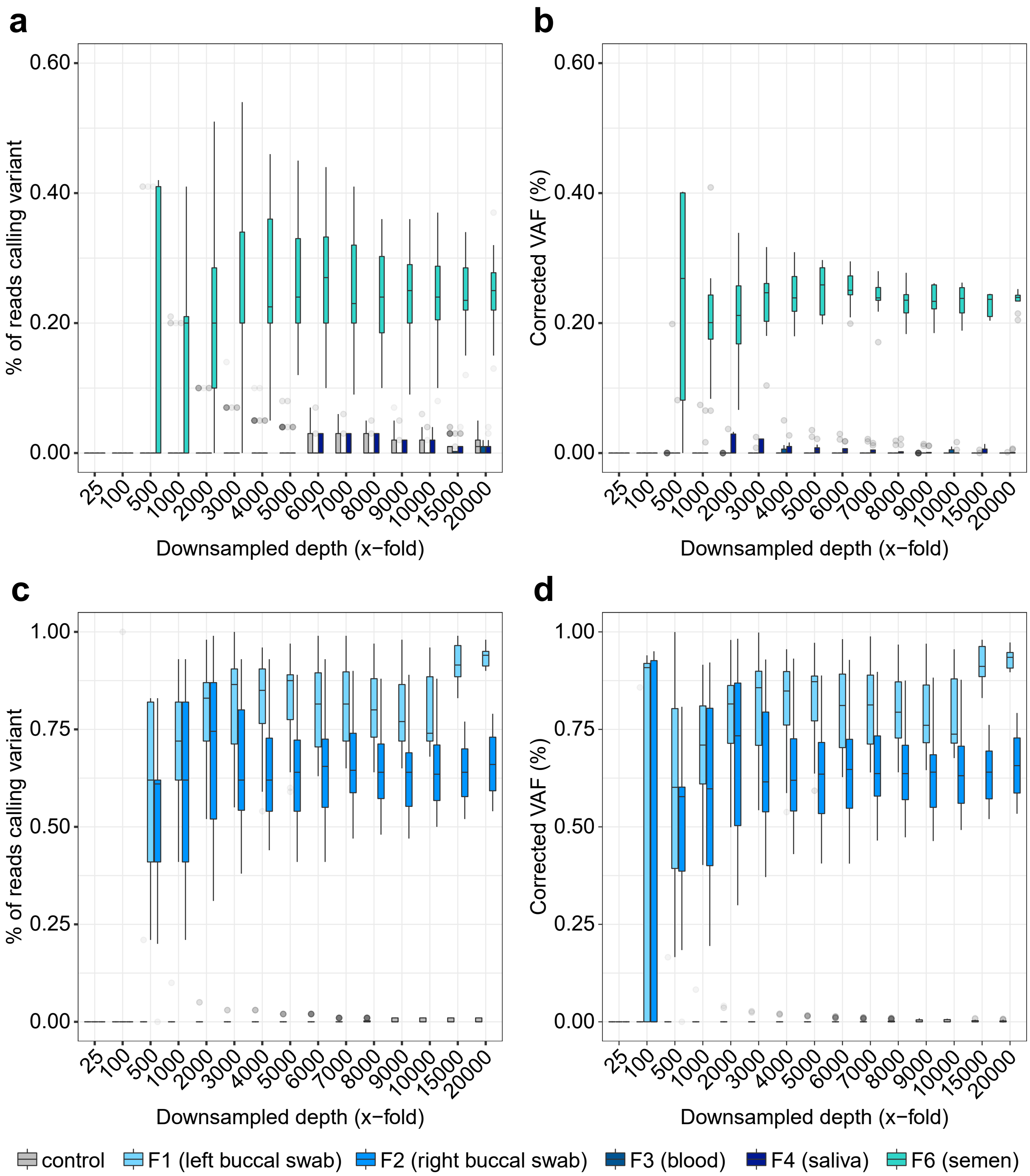


**Supplementary Fig. S3: Downsampled Deep-NGS results for selected biological samples in families FAM27 (A-B) and FAM34 (C-D).** The percentage of mutant reads (A and C) and the corrected variant allele frequency (VAF) (B and D) for different tissue samples (buccal swab, blood, saliva and semen, visualized by different colors as indicated on the Figure) and three unrelated controls, after downsampling to a range of sequencing depths (indicated on the x-axis). Each sample and each control were amplified and sequenced as independent replicates, with each of the triplicates randomly downsampled ten times to each depth (See Supplementary Note 3 for details). Boxes represent the interquartile range of each variable, with midlines representing the median. Upper and lower whiskers extend, respectively, to the largest and smallest values no further than 1.5x the interquartile range. Data beyond the ends of each whisker are outliers and plotted individually, with more remote outliers increasingly transparent. For ease of visualization, the color scheme and sample abbreviations are as in Supplementary Fig. S2.

## <u>Supplementary Note 1</u>: Stratification of DNMs into 7 categories

To determine the relative proportion of cases in the seven categories of *de novo* mutation (DNM) origin presented in Figure 1, we used the data described in Rahbari *et al* (2016)[1]. In this study, a total of 768 DNMs were identified across whole-genome sequencing (WGS) data (average depth: 25x) for three multi-sibling families comprising 4-5 individuals and their parents. Among these, 399 DNMs could be haplotyped with respect to a nearby inherited polymorphism and allowed the parent-of-origin to be determined, demonstrating that 78% (311/399) of DNMs were paternal in origin, a result consistent with other studies[2-5].

The proportion of DNMs that were likely to have originated through parental mosaicism could be determined via two different approaches:

(a) If mosaic mutations are present in multiple parental gametes, recurrence in siblings may be observed. In the Rahbari study[1], ten validated DNMs were shared by at least two siblings of the same family, providing an estimate of any germline mutation being shared by at least two siblings as 1.3% (10/768). However, some of the mosaic variants were shared by more than two siblings in the family. Hence, another, more exact, way to estimate the observed recurrence of DNMs in these 3 families, proposed by Rahbari *et al.* in Supplementary Data S2, described a total of 2850 sib-sib comparisons (sum of number of DNMs multiplied by the number of other siblings in that family, for each given DNM) and 34 instances of shared DNMs between sib pairs, leading to a figure of 1.19%, the probability that a given DNM is also present in another sibling (i.e. the recurrence risk for a DNM that has already been observed in one individual of the family, conditional on parental blood VAFs being not detected at a depth of ~25x, a sequencing depth estimated to have a sensitivity ~10%). Overall, this genome-wide estimate of DNM recurrence, although based on a small number of multi-sibling families, provides a figure in line with the empirical 1-2% population recurrence risk[6,7].

(b) A mosaic (parental) origin for validated DNMs was also sought by deep-sequencing (average 567x) of parental blood DNA (note, blood was the only tissue available for this study)[1]. This approach showed that 25 DNMs observed in children were in fact detectable at low VAF (observed average VAF of 3.2% (range: 0.6-10.2%)). Of note, the pipeline used for calling the 768 DNMs in the three families conservatively balanced the need for sensitivity and specificity in this dataset, as demonstrated by the low VAFs observed by deep-sequencing for the 25 mosaic mutations in parents which were all ≤10.2%. After correction for incomplete power to detect parental mosaics at VAF < 1%, these data suggested that ~4.2% of DNMs observed in children are present in the somatic tissues (mixed mosaicism) of one of the parents. As expected for these early mutational events,

which arise well in advance of sex determination in humans (that takes place at ~6 weeks of embryonic life)[8,9], they were approximately equally distributed among the originating parent (paternal:maternal ratio = 9:16), a result consistent with other estimates[4]. This implies that, overall, paternal (Category C) and maternal (Category F) mixed mosaicism are each responsible for ~2% of the DNMs occurring in a child (Fig. 1).

By comparing the two approaches above, the contribution of confined gonadal mosaicism (Categories B and E) can be deduced, as among the ten recurrent DNMs, six could also be detected in the blood of one of the parents (i.e. mixed mosaic) and four were undetectable at ~500x (i.e. indicative of confined gonadal mosaicism). From this, it can be inferred that the proportion of mixed and gonadal mosaic mutations are likely comparable and corresponds to ~4% each, split equally between the two parents, meaning that each of the Categories B, C, E and F corresponds to ~2% of DNMs (Fig. 1).

The proportion of DNMs belonging to Category G (high-level VAF post-zygotic mutation having occurred after fertilization in the proband) is based on data from Acuna-Hidalgo *et al*. (2015)[10] who analysed a set of 107 DNMs from 50 parent-offspring trios, using four different sequencing techniques, and showed that seven (6.5%) of these apparent DNMs had VAF significantly different to the 50:50 ratio expected for heterozygous germline variants. These post-zygotic Category G variants are expected to arise equally on the maternal and paternally derived chromosomes.

Taking all these data into consideration allows us to estimate the representation of the different mosaic events into each category A-G as presented on Figure 1. The contribution of Categories A and D (one-off events of paternal or maternal origin, respectively) can be deduced, given that globally 78% of DNMs are paternal (and 22% are maternal) in origin.

Overall, these data suggest that ~8% of all candidate DNMs identified in a proband are in fact present as mosaic in the parental germline (Categories B-C or E-F) and represent a recurrence risk in a future pregnancy. Of note, given that 78% of DNMs are paternal in origin this also implies that a mosaic origin is expected 3-4x more frequently for a DNM of maternal origin than for paternally-derived DNM.

Although the genome-wide estimates of the occurrence of different categories of DNM used in Figure 1 are based on studies[1,6] of relatively small sizes, these estimates are concordant with more recent data[4,5]. The 33 large (~8 siblings) three-generation families analyzed in the Sasani *et al.* study[4] allowed them to show that 1.3% (303/23,386) of DNM sites are shared by one or more (up to 5) siblings of the same family. To refine this estimate, we used the Sasani dataset and performed the

same analysis as that described in Supplementary Data S2 of Rahbari[1] (see above) to establish the proportion of a DNM being shared amongst any two siblings (conditioned on the fact that the DNM was not detected in parental blood at ~30x sequencing depth). We considered the 23,386 DNMs called in the multi-sibling third generation and the observed 720 shared mosaic events (across 303 unique sites). These represent a total of 1156 sharing events (sum across individuals of number of shared occurrences of a DNM [i.e. for each shared mutation, count how many individuals (other than themselves in this family) carried this DNM]) from a total 221,569 sib-sib comparisons (sum across individuals of number of sibs [not including themselves] * [number of DNMs]), which provides an observed recurrence between any two siblings of 0.52%, a factor ~2 lower than that from the analysis of the three families described in Rahbari[1]. Interestingly, the 1.19% average estimate from the Rahbari study[1] was mainly contributed by one of the three families analysed with individual values of 0% (0/780), 0.3% (2/706) and 2.3% (32/1364).

Of note, this generic 1-2% figure is an empiric estimate, based on recurrence observed for different clinical disorders – often reported as case studies – that were ascertained using a variety of methodologies and for which it is difficult to find a reliable data source[6,7]. Moreover, depending on the disorders and associated mutated genes, the risk of mosaic presentation may be lower (i.e., paternal age-effect disorders, such as Apert syndrome or achondroplasia[11]) or higher (i.e., Duchenne and Becker muscular dystrophy[12], osteogenesis imperfecta[13], Dravet syndrome[14]) than the population risk for non-pathogenic mutations.

Another factor to be considered when analyzing WGS data is that identifying de novo variant calls from family trio data will depend on the methods and the filters used for their identification in the first instance, which likely will differ between clinical and research settings. Recently, Bergeron *et al*. showed that analysis of the same trio dataset by five different research groups estimated DNM rates that varied greatly (by a factor 2) depending on the methods and approaches used to call variants and aligned reads[15]. This comparative study illustrates some of the challenges faced in the clinic for systematic detection of DNMs (and identification of low level mosaicism in parental samples) from data generated through different methodologies.

Finally, the unique three-generation design of the Sasani study[4] also provides an estimate of the proportion of high-level post-zygotic mosaic cases (Cat G) caused by very early events (before the soma-germline split) in the second-parental generation. These mosaic DNMs will exhibit high VAFs that are difficult to distinguish from the 50:50 true heterozygous germline DNM (such as those of Category G in our study). By looking at the transmitted haplotype around the DNM called in the second-parental generation within the third-generation probands, the authors can show that 9.3%

of DNMs (475/5017 DNMs) observed in the second generation are, in fact, post-zygotic mosaic in these individuals. This also allows them to confirm that this process is sex-independent as each parent contributes equally to this tally (paternal:maternal ratio = 249:226).

**<u>Supplementary Note 2</u>: Design and results for assessment of mosaicism in two families with the larger indel DNMs (FAM12b and FAM54)**

For FAM12, one of the proband's DNM was a 44 bp deletion in *MECP2* (FAM12b) for which we designed a mutation-specific PCR assay using a reverse primer encompassing the deletion junction with the 5' sequence starting at g.X:154,030,684 (GRCh38 genome build) and the sequence 5'-CTGCTCCCACCCCTGCCtCa**CTG**-3', where t and a represent introduced mismatches and the sequence in bold (CTG) represents g.X:154,030,618_154,030,620, located on the other side of the deletion. PCR in combination with a forward primer (g.X:154,030,385-154,030,404) (Tm = 66 °C) was able to amplify specifically the mutant fragment containing the 44 bp deletion and the presence of the mutant fragment (256 bp) was assessed on a 3% agarose gel. Serial dilution of the proband gDNA to levels down to 1:650 (using carrier DNA), showed robust amplification of the mutation-specific fragment at this dilution, demonstrating assay sensitivity to levels below 0.08% (1:1,300). In parallel, a control PCR flanking the deletion (using the following primers: Forward: g.X:154,030,578-154,030,597 and Reverse: g.X:154,030,796-154,030,777) was performed to ensure robust amplification of all the family samples tested and to assess the presence of the two alleles (generating fragments of 175 bp (mutant) vs. 219 bp (WT)) in the expected ratio in the proband's samples. Consequently, there is no evidence suggestive of a post-zygotic mosaic event in the proband. Using the mutation-specific PCR assay, a robust amplification of the 256 bp band was observed for all the three proband samples (left and right buccal swabs and blood), but no amplification of the parental samples (blood, saliva, buccal swabs, urine and semen) was detected; therefore, no evidence of mosaicism was detected in any of the parental samples.

For FAM54 the DNM was a 35 bp duplication in *MAGEL2* and two PCR assays were used to screen all the family samples and assess the results on agarose gels. First, a genomic region flanking the duplication (using the forward primer: g.15:23,645,351_23,645,372 and reverse primer: g.15:23,645,608_23,645,585) was defined to ensure robust amplification of all the family samples tested and to assess the presence of the two alleles. By inspection, the two fragments (293 bp (mutant) vs. 258 bp (WT)) were present in the expected ratio in the proband (no evidence to suggest post-zygotic mosaicism), while a single band of 258 bp (and no mutant 293 bp) was observed in the parental or control samples. We then designed an allele-specific PCR assay (using a reverse primer encompassing the duplication junction with 5' sequence starting at g.15:23,645,498 (5'-CGCATGATCTTTGCTGC**AGG**-3', where the sequence in bold (AGG) represents g.15:23,645,516-23,645,514, located specifically within the duplication) in combination with the forward primer described above, able to amplify specifically the mutant fragment containing the 35 bp duplication.

The presence/absence of the mutant band (183 bp) was assessed for all the family samples on a 3% agarose gel. Moreover, serial dilution of the proband gDNA to levels down to 1:650 (using carrier DNA), showed robust amplification of the mutation-specific fragment (183 bp) at this dilution, demonstrating assay sensitivity to mutation levels below 0.08% (1:1,300). Using the mutation-specific PCR assay, robust amplification of the 183 bp band was observed for all the three proband samples (blood, left and right mouth swabs), but no amplification of the parental samples (blood, saliva, mouth swabs, urine or paternal semen) or in control samples was detected; therefore, we concluded that no evidence of mosaicism was found in any of the parental samples.

## Supplementary Note 3: Sensitivity of the Deep-NGS assay for low VAF quantification

Measuring low VAFs is technically challenging and requires sensitive methods that minimize the occurrence of false positive and false negative results. Many factors influence the likelihood of calling a variant present at low VAF using targeted NGS, including PCR amplification protocol, depth of sequencing, the specific genomic context of the nucleotide of interest, the type of variant substitution, and both the data analysis pipelines and parameters used for variant calling.[16] To maximise the sensitivity of the Deep-NGS assay in this study, we used high fidelity (proof-reading) polymerases and performed all PCR reactions in triplicates (technical replicates) to reduce the risk of false positive calls due to PCR errors. We used the Illumina MiSeq platform to sequence the triplicate samples and only considered high quality base calls (Phred quality score >30) when variant calling using Amplimap (see Methods).

To identify mosaic VAFs at levels ≤1% and reduce the risk of false negative low VAF calls, we aimed to sequence each triplicate amplicon at a depth >5,000x. Furthermore, to account for the background noise caused by the genomic context and substitution type of the DNM (which varies for each custom Deep-NGS assay), we also made use of the reads generated from the three unrelated control samples to correct the raw VAF values obtained for each biological sample (see Methods).

To illustrate the benefits of high sequencing depths and the VAF correction strategy, we performed a downsampling analysis of the biological samples from the mosaic parent for two families for which individual replicates had been sequenced at depths >20,000x each. These families were FAM27 (the lowest corrected VAF for the DNM is found in the semen sample F6 and measured as corrected VAF = 0.23%) and FAM34 (the lowest corrected VAFs for the DNM are in the buccal brush samples F1 and F2, measured as 0.85% and 0.61% respectively; see Supplementary Data S2 and Supplementary Fig. S3).

As described in the main text, Deep-NGS data were originally analyzed using Amplimap v0.4.9 [17] to obtain both the VAF of each family-specific mutation and the total count of >Q30 bases at the corresponding genomic position (GRCh38.p12) in each PCR replicate and sample. For each replicate and sample, Amplimap produces a BAM. Using samtools 'depth' with parameters -d 0 -r, we first determined the median sequencing depth across all positions of the target region. We then downsampled each BAM to x-fold coverage per base, where x was 25, 100, 500, and 1000, every multiple of 1000 to 10,000, plus 15,000 and 20,000 (i.e. downsampling to 15 different depths). Downsampling was performed 10 times per depth using Picard Tools v2.27.2 DownsampleSam (http://broadinstitute.github.io/picard/) with parameters --STRATEGY HighAccuracy and --P, where P

(the probability of retaining a given read when iterating through the BAM) was equal to desired fold-depth / median sequencing depth. Samples where P was either not a number or a number > 1 (indicative of a failed, low-, or zero-depth sample) were discarded. DownsampleSam applies a downsampling algorithm to retain only a deterministically random subset of reads, with the probability of any given read being included in this subset equal to P. The 'strategy' parameter provides a range of options tailored to the available memory and size of the input BAM. The 'HighAccuracy' strategy attempts to output a proportion of reads as close to the requested proportion as possible, although is the most memory-intensive. To facilitate reproducibility, seeds were not randomly generated but manually assigned: 123, 456, 789, 234, 567, 891, 321, 654, 987, and 432.

Finally, for each PCR replicate, sample, fold-depth and seed, we re-ran Amplimap 'pileups' as previously described but in 'mapped_bams_in' mode. The workflow for this analysis is available at github.com/sjbush/pregcare.[18]

The results are illustrated in Supplementary Figure S3 and plot both the % of mutant reads (Supplementary Fig. S3A&C) and the corrected VAFs (Supplementary Fig. S3B&D). For both families, this analysis shows that, as expected, low-frequency VAFs cannot realistically be called at standard (30-100x) sequencing depths and that background noise – the % of mutant reads in the controls that (falsely) call the variant – increases with depth (Supplementary Figs S3A and S3C). While this is mitigated by the VAF correction strategy (Supplementary Figs S3B and S3D), the data also show that, in the case of FAM27, a minimal cutoff depth of ~3000-4000x is necessary to ensure that the VAF for sample F6 (0.23%) is reliably above the assay's noise.

**Supplementary Note 4: Successes and failures in resolving haplotypes using long-read sequencing (MinION platform from ONT)**

In total, 50 family trios were sequenced using locus-specific long-read sequencing on the ONT platform. These included 47 families without evidence of mosaicism as presented in the main text and three families in which we detected mosaicism and were included as controls (FAM17 (Cat F), FAM27 (Cat B) and FAM34 (Cat C)). In this series, DNMs consisted of 37 SNVs and 13 indels. Overall, we were able to resolve the phase for 41 DNMs.

The workflow used to haplotype DNMs is available at github.com/sjbush/pregcare[18] and sequentially implements the programs Medaka and mpileup. In brief, if Medaka fails to resolve phase, the workflow will attempt to do so using pileup. Should phase not be resolved using pileup, manual phasing is attempted.

More specifically, Medaka resolved the inheritance pattern for 21 of the SNVs (56.8% of the total SNVs) but none of the indels. Poorer performance with indels was likely due to the relatively error-prone nature of ONT sequencing data[19]. Furthermore, according to ref.[20], Medaka first predicts SNVs from unphased reads and then uses WhatsHap[21] to phase them. Indel calling – technically more challenging – was performed later, and only using reads already phased, a subset of the total. Through data curation, failures to resolve the inheritance of the SNVs with Medaka (16/37) could be attributed to the DNM not being called in the child (six families), the DNM being called but not assigned a phase set (five families), and to the set of in-phase SNPs being either identical in both parents (four families) or considered by Medaka to be low-quality calls (one family). Failures to resolve the inheritance of indels with Medaka could be attributed to the DNM not being called in the child (12 families) and the DNM being called but not assigned a phase set (one family). Of the 29 families for which inheritance was not resolved using Medaka, we resolved inheritance using pileup in 15 cases (see Methods and Supplementary Data S3A & S3B). For one family (FAM02), the informative SNP was an indel and the DNM was phased manually. For a further three families (FAM11, FAM38 and FAM67), the phase was resolved manually using a '2-SNP' design; i.e. we first identified a SNP that was heterozygous in all three family members and used another discriminant SNP heterozygous in one of the parents to assign the phase of each allele to their parent-of-origin (detailed in Supplementary Data S3B).

For the nine remaining families (5 SNVs and 4 indels), we were unable to resolve the phase of the DNM. A key requirement for haplotype phasing of DNMs is the presence of a heterozygous SNP in the vicinity of the DNM in the proband, in order to distinguish the two parental alleles. In all nine

families, no heterozygous SNP could be identified near the DNM in the proband, despite sequencing a total of >10 kb around the DNM (and >20 kb in 7 families; see Supplementary Data S3A). In theory, these DNMs can be haplotyped, but this will require further extension of the size of the genomic region interrogated. Implementation of novel and/or ultra long-read technologies, such as those exploited by the Telomere-to-Telomere (T2T) sequencing approach, will undoubtedly improve our ability to phase DNMs in the future[22]. For example in a recent study, Noyes *et al.*[23] showed that 194/195 DNMs identified in a family quartet could be assigned to their parent-of-origin, using informative SNPs extending 20 kb on either site of the DNM location.

## Supplementary Note 5: Allele-specific PCR for haplotyping the DNM in *AHDC1* in FAM38

For FAM38, the long-read sequencing performed on the ONT platform did not allow unambiguous discrimination of the two alleles at the DNM position g.1:27548981 (deletion G) in the *AHDC1* gene in the proband due to a homopolymeric region (GGGG (mutant) vs. GGGGG (REF)). Using the ONT data, we identified a SNP (rs2076457) that was heterozygous in the three members of the family. We could establish the parent-of-origin of each of the two rs2076457 alleles (paternal A and maternal C) in the proband by using the phase in respect to another discriminant SNP (rs113173951G/A), which was heterozygous in the mother only. Hence, the rs2076457 SNP was used for haplotyping the *de novo* mutation by performing an allele-specific PCR amplification followed by dideoxy-sequencing. To amplify only the maternally-derived rs2076457C allele in the proband, the reverse primer 5'-CAGCCGCTGGGGTCGGGGGCaCG-3', where a represents a deliberate mismatch and G is rs2076457C allele-specific, was combined with a forward primer (g.1:27,548,900_27,548,920), to generate a fragment of ~1.1 kb. Dideoxy-sequencing of this PCR fragment revealed that the maternally-derived rs2076457C allele is in phase with the *de novo* mutation (g.1:27,548,981delG) (see Supplementary Data S3B).

**<u>Supplementary Note 6</u>: Estimating the recurrence risk associated with mosaicism using WGS data from multi-sibling families and sperm WGS studies**

In what follows, we define recurrence risk (RR) to be the probability that a given DNM observed in a proband is present in the next offspring from the same couple. Notably, this means the estimates of recurrence risk obtained below would be inappropriate if sequencing results from another offspring were known. We further condition our estimates on the fact that the DNM has been called in the proband (i.e. HET) and was not called (i.e. HOM REF) from both parents, given that they had been sequenced using NGS at ~25-30x depth. Importantly, there is relatively limited sensitivity of variant calling using routine NGS, where calling a mosaic VAF = 15% would require detection, and filtering in, of only 3-4 mutant reads in a parent sequenced at 25x[24]; this sensitivity is similar to the 15-20% limit of detection of dideoxy-sequencing[25].

We used the estimates of the proportion of DNMs belonging to each Category A through G, as described on Fig. 1 and in Supplementary Note 1.

Next, we used data from Yang *et al.*[26] that describes deep (200-300x) whole genome sequences (WGS) of paired blood and sperm samples from 17 men, to derive estimates of the distribution of gonadal variant allele frequencies (VAFs) for mosaic variants, conditional on them being either detected in both sperm and blood (i.e., mixed mosaic corresponding to Category (Cat) C and by inference Cat F), or just being mosaic in sperm (i.e., confined gonadal mosaic corresponding to Cat B and by inference Cat E). Of note, in the absence of direct estimates of female gonadal mosaic frequencies, given that these are very early events, we assume that the distributions of VAFs are the same for Categories B and E, and likewise for Categories C and F (see also Supplementary Note 1 and Supplementary Fig. S1).

We downloaded Data S1 of Yang et *al.*[26], and for gonadal tissue only (Categories B (and E)), used entries from COHORT = “Young Age” or “Advanced Age”; SET_SPERM_ONLY = 1, and used column MAF_SPERM_A. For mixed mosaic (Categories C (and F)), we used entries from COHORT = “Young Age” or “Advanced Age”; SET_BOTH_MOSAIC = 1, and again used column MAF_SPERM_A. We further stratified the mosaic data by restricting them to MAF_BLOOD < 0.15 as an approximation, as variants with higher mosaic VAFs in blood are less likely to be assigned as DNMs (for which the parental sample must be called as HOM REF) in WGS at ~25-30x, which would violate the earlier assumption. We also performed the calculations without this MAF_BLOOD < 0.15 restriction, to model an upper bound of recurrence when blood is not used for WGS and/or VAFs are very variable

in different somatic tissues. Notably, we did not include the data from COHORT = "ASD" as these samples were sequenced at a lower depth (200x vs. 300x)[16].

We obtained an estimate of the average VAF for gonadal (Cat B and Cat E) and mixed mosaic (Cat C and Cat F) as follows:

For SPERM-only variants (n = 418): $VAF_{Sperm}$ = 3.01%

For MIXED mosaic (all variants) (n = 190): $VAF_{Sperm}$ = 8.39%

For MIXED mosaic (for variants with $VAF_{Blood}$ <0.15) (n = 151): $VAF_{Sperm}$ = 4.89%

To calculate the recurrence risk, we make the assumption that each DNM in a given category must be of a given "type", and that each DNM of the same type has the same underlying VAF in the originating parent's gonadal tissue. We model each observation in the Yang data as akin to a DNM type, with $VAF_{sperm}$ as the probability of observing the non-reference (ALT) allele for that DNM type. Using Bayes' Theorem, we model that the probability a DNM is type i (i.e. when observed in a given category), as being proportional to the VAF of a DNM of type i from that category. Specifically, here we model that the probability of an observed DNM of a given category is type i (i = 1 through the length of the Yang $VAF_{sperm}$ data for that category) is the frequency of that type as specified in the Yang data, divided by the total frequency for that category (as given in the Yang data).

We therefore calculate the recurrence risk (RR) as follows: where *R* is the event of recurrence (i.e. a ALT allele for the observed DNM is present in the next offspring at the same genomic site); DNM obs is the event of observing a DNM at an otherwise arbitrary site. Then we have that: P(Cat = *X* | DNM obs) is the previously described probability of the observed DNM belonging to categories A through F; P(Type = *i* | Cat = *X*, DNM obs) is the previously described probability that an observed DNM of category *X* has the property of type *i* (i.e. has true underlying gonadal allele frequency of type *i*); and P(*R* | Type = *i*, Cat = *X*, DNM obs) is the probability of recurrence given an observed DNM of category *X* and type *i*, which has gonadal allele frequency of type *i*.

$$(1) \qquad \mathrm{RR} = \mathrm{P}(R \mid \mathrm{DNM\ obs})$$

$$= \sum_{X\ in\ A:F} \mathrm{P}(R, \mathrm{Cat} = X \mid \mathrm{DNM\ obs})$$

$$= \sum_{X\ in\ A:F} \mathrm{P}(R \mid \mathrm{Cat} = X, \mathrm{DNM\ obs})\ \mathrm{P}(\mathrm{Cat} = X \mid \mathrm{DNM\ obs})$$

$$= \sum_{X\ in\ A:F} \sum_{type\ i} \mathrm{P}(R, \mathrm{Type} = i \mid \mathrm{Cat} = X, \mathrm{DNM\ obs})\ \mathrm{P}(\mathrm{Cat} = X \mid \mathrm{DNM\ obs})$$

$$= \sum_{X \text{ in } A:F} \sum_{type\ i} \mathrm{P}(R \mid \mathrm{Type} = i, \mathrm{Cat} = X, \mathrm{DNM\ obs})\, \mathrm{P}(\mathrm{Type} = i \mid \mathrm{Cat} = X, \mathrm{DNM\ obs}) \mathrm{P}(\mathrm{Cat} = X \mid \mathrm{DNM\ obs})$$

Together, this allows us to estimate the recurrence risks as presented in the table below, for the overall population risk, as well as when the DNM belongs to a subset of categories - maternal origin without suspicion of somatic mosaicism (D/E), unresolved parent-of-origin, without suspicion of mosaicism in parental tissues (A/D/E), or mosaic cases (mixed C/F, or gonadal B/E). Note, as explained above, we report results both when restricting Yang mosaic sperm variants with $VAF_{Blood}$ <0.15, which generally is likely to best represent the situation most families would fall into after routine clinical testing, as well as without this cut-off.

| **Category** | **RR % ($VAF_{Blood}$ <0.15)** | **RR % (all)** |
|---|---|---|
| Overall | 0.58 | 0.89 |
| D or E | 0.49 | 0.49 |
| A, D or E | 0.095 | 0.095 |
| C or F | 10.27 | 17.95 |
| B or E | 4.18 | 4.18 |

For comparison, we note that Rahbari *et al*.[1] estimated an overall RR = 1.2% in their Supplementary Data S2. By performing a similar calculation using substantially more data from Sasani *et al*.[4], we obtain an estimate of 0.52% (see Supplementary Note 1 for details), which is similar to our estimate of RR for $VAF_{sperm}$ <0.15 of 0.58% above.

From these data, it also follows that screening couples by deep-sequencing of multiple somatic tissues to detect the cases of parental mixed mosaicism (C or F) combined with sperm analysis to identify cases of paternal gonadal mosaicism (B), offers the possibility to reduce the remaining RR for the other couples (Cat A, D or E) to ~ 0.1%, representing approximately a 10-fold risk reduction from the starting overall population risk.

**Estimating confidence intervals on population risk estimates**

We estimated confidence intervals for the above estimates using bootstrapping. For each bootstrapping replicate, we re-sampled the variant allele frequency distributions from Yang *et al.*[16].

To form confidence intervals (CIs), we performed 100,000 bootstrap replicates, and took the $2.5^{th}$ and $97.5^{th}$ percentiles. This generated the following estimates and confidence intervals (in %)

| Category | RR % ($VAF_{Blood}$ <0.15) | RR % (all) |
|---|---|---|
| Overall | 0.58 (0.5, 0.64) | 0.88 (0.8, 0.96) |
| D or E | 0.49 (0.43, 0.57) | 0.49 (0.43, 0.57) |
| A, D or E | 0.09 (0.08, 0.11) | 0.09 (0.08, 0.11) |
| C or F | 10.23 (8.5, 11.81) | 17.91 (15.8, 19.83) |
| B or E | 4.16 (3.62, 4.82) | 4.16 (3.62, 4.82) |

We note that these CIs are likely insufficiently conservative (i.e. too narrow) as we are not correcting for intra *vs.* inter family variation in this bootstrapping, and we do not incorporate uncertainty in knowledge of the probability of the different categories.

We also note that the above are CIs, and as such, interval estimators that should contain the true underlying parameter with the appropriate confidence (here 95%). As new studies similar to the Yang *et al.*[16] dataset become available to better estimate CIs, these intervals would shrink to eventually reach a zero width. However, they do not reflect the fact that VAF varies across different families within the same category, resulting in significantly different levels of individual risk (i.e. when the VAF in gonadal tissue for a given DNM differs significantly from the sub-group average). We therefore calculated in each bootstrapping replicate from each of the sub-categories defined in the Table above, the recurrence risk corresponding to the 2.5th and 97.5th percentile of risk. We note that these estimates are not confidence intervals. Averaging these across bootstrapping runs yields the following (in %).

| Category | RR % ($VAF_{Blood}$ <0.15) | | RR % (all) | |
|---|---|---|---|---|
| | **$2.5^{th}$ ile** | **$97.5^{th}$ ile** | **$2.5^{th}$ ile** | **$97.5^{th}$ ile** |
| D or E | 0.0 | 5.0 | 0.00 | 5.0 |
| A, D or E | 0.0 | 0.0 | 0.00 | 0.00 |
| C or F | 1.0 | 20.7 | 1.5 | 33.5 |
| B or E | 1.4 | 13.5 | 1.4 | 13.5 |

Overall, the RR estimates based on combining data from multi-sibling and sperm WGS studies are similar but slightly lower than the 1-2% population risk quoted in the clinic.

**Supplementary Note 7: Choice of biological samples to analyze for mosaicism.**

Our data show that there is a clear benefit from collecting multiple tissue samples to increase sensitivity for ascertaining instances of occult mosaicism. It would be valuable to know whether there is a particular somatic tissue that provides a better surrogate for the germline, especially for the maternal mosaic cases, where there is no easy access to oocytes. This can be addressed by studies of paternal mosaic samples (Category C). Although we observed substantial VAF variation between somatic samples (Figure 3, Supplementary Fig. S2), there was no clear surrogate yielding values similar to the germline VAF, consistent with the fact that during early embryogenesis, cell populations are subject to bottlenecks and differential lineage commitments leading to considerable variation and stochasticity in cellular representation across tissues.[5,27] Similar results have been reported in other studies that analyzed several different somatic tissues.[14,28-30] Overall, reliance on assessment of a single tissue (blood) risks missing some mixed mosaics harboring low mutation levels (or high level post-zygotic mosaicism in the proband). Of the other somatic tissues we sampled, we found that saliva tended to reflect the results from blood[24,31] but occasionally exhibited a higher background of apparent mutant sequences that can bias low VAF interpretation, likely reflecting the fact that ~70% of saliva DNA is derived from white blood cells, while the remaining fraction contains bacterial and/or other genomes (potentially including that of other family members, including the proband)[32]. Unlike urine, which often yielded poor amounts of DNA, especially for the paternal sample, buccal brushings are easy to collect (including from children) and store, and contain cells of a different embryological origin to blood, which often yielded informative data.

## Supplementary References


1. Rahbari, R.*, et al.* Timing, rates and spectra of human germline mutation. *Nat Genet* **48**, 126-133 (2016).
2. Kong, A.*, et al.* Rate of de novo mutations and the importance of father's age to disease risk. *Nature* **488**, 471-475 (2012).
3. Jonsson, H.*, et al.* Parental influence on human germline de novo mutations in 1,548 trios from Iceland. *Nature* **549**, 519-522 (2017).
4. Sasani, T.A.*, et al.* Large, three-generation human families reveal post-zygotic mosaicism and variability in germline mutation accumulation. *Elife* **8**, e46922 (2019).
5. Jonsson, H.*, et al.* Differences between germline genomes of monozygotic twins. *Nat Genet* **53**, 27-34 (2021).
6. Zlotogora, J. Germ line mosaicism. *Hum Genet* **102**, 381-386 (1998).
7. Cook, C.B.*, et al.* Somatic mosaicism detected by genome-wide sequencing in 500 parent-child trios with suspected genetic disease: clinical and genetic counseling implications. *Cold Spring Harb Mol Case Stud* **7**, a006125 (2021).
8. Nef, S., Stevant, I. & Greenfield, A. Characterizing the bipotential mammalian gonad. *Curr Top Dev Biol* **134**, 167-194 (2019).
9. Saitou, M. & Hayashi, K. Mammalian in vitro gametogenesis. *Science* **374**, eaaz6830 (2021).
10. Acuna-Hidalgo, R.*, et al.* Post-zygotic Point Mutations Are an Underrecognized Source of De Novo Genomic Variation. *Am J Hum Genet* **97**, 67-74 (2015).
11. Wilkie, A.O.M. & Goriely, A. Gonadal mosaicism and non-invasive prenatal diagnosis for 'reassurance' in sporadic paternal age effect (PAE) disorders. *Prenat Diagn* **37**, 946-948 (2017).
12. Helderman-van den Enden, A.T.*, et al.* Recurrence risk due to germ line mosaicism: Duchenne and Becker muscular dystrophy. *Clin Genet* **75**, 465-472 (2009).
13. Pyott, S.M.*, et al.* Recurrence of perinatal lethal osteogenesis imperfecta in sibships: parsing the risk between parental mosaicism for dominant mutations and autosomal recessive inheritance. *Genet Med* **13**, 125-130 (2011).
14. Yang, X.*, et al.* Genomic mosaicism in paternal sperm and multiple parental tissues in a Dravet syndrome cohort. *Sci Rep* **7**, 15677 (2017).
15. Bergeron, L.A.*, et al.* The Mutationathon highlights the importance of reaching standardization in estimates of pedigree-based germline mutation rates. *Elife* **11**(2022).
16. Petrackova, A.*, et al.* Standardization of Sequencing Coverage Depth in NGS: Recommendation for Detection of Clonal and Subclonal Mutations in Cancer Diagnostics. *Front Oncol* **9**, 851 (2019).
17. Koelling, N.*, et al.* amplimap: a versatile tool to process and analyze targeted NGS data. *Bioinformatics* **35**, 5349-5350 (2019).
18. Bush, S.J, Thibaut, L. Personalized recurrence risk assessment following the birth of a child with a pathogenic de novo mutation. Pregcare, https://zenodo.org/record/7501575 (2023).
19. Goodwin, S.*, et al.* Oxford Nanopore sequencing, hybrid error correction, and de novo assembly of a eukaryotic genome. *Genome Research* **25**, 1750-1756 (2015).
20. Ahsan, M.U., Liu, Q., Fang, L. & Wang, K. NanoCaller for accurate detection of SNPs and indels in difficult-to-map regions from long-read sequencing by haplotype-aware deep neural networks. *Genome Biology* **22**, 261 (2021).
21. Patterson, M.*, et al.* WhatsHap: Weighted Haplotype Assembly for Future-Generation Sequencing Reads. *Journal of Computational Biology* **22**, 498-509 (2015).
22. Akbari, V.*, et al.* Parent-of-origin detection and chromosome-scale haplotyping using long-read DNA methylation sequencing and Strand-seq. *BioRxiv* doi: https://doi.org/10.1101/2022.05.24.493320 (2022).

23. Noyes, M.D., *et al.* Familial long-read sequencing increases yield of de novo mutations. *Am J Hum Genet* **109**, 631-646 (2022).
24. Gambin, T., *et al.* Low-level parental somatic mosaic SNVs in exomes from a large cohort of trios with diverse suspected Mendelian conditions. *Genet Med* **22**, 1768-1776 (2020).
25. Tsiatis, A.C., *et al.* Comparison of Sanger sequencing, pyrosequencing, and melting curve analysis for the detection of KRAS mutations: diagnostic and clinical implications. *J Mol Diagn* **12**, 425-432 (2010).
26. Yang, X., *et al.* Developmental and temporal characteristics of clonal sperm mosaicism. *Cell* **184**, 4772-4783 e4715 (2021).
27. Coorens, T.H.H., *et al.* Extensive phylogenies of human development inferred from somatic mutations. *Nature* **597**, 387-392 (2021).
28. Huang, A.Y., *et al.* Postzygotic single-nucleotide mosaicisms in whole-genome sequences of clinically unremarkable individuals. *Cell Res* **24**, 1311-1327 (2014).
29. Zhang, Q., *et al.* Genomic mosaicism in the pathogenesis and inheritance of a Rett syndrome cohort. *Genet Med* **21**, 1330-1338 (2019).
30. Yang, X., *et al.* ATP1A3 mosaicism in families with alternating hemiplegia of childhood. *Clin Genet* **96**, 43-52 (2019).
31. Wright, C.F., *et al.* Clinically-relevant postzygotic mosaicism in parents and children with developmental disorders in trio exome sequencing data. *Nat Commun* **10**, 2985 (2019).
32. Samson, C.A., Whitford, W., Snell, R.G., Jacobsen, J.C. & Lehnert, K. Contaminating DNA in human saliva alters the detection of variants from whole genome sequencing. *Sci Rep* **10**, 19255 (2020).
