# Supplementary material for: Personalized recurrence risk assessment following the birth of a child with a pathogenic de novo mutation
Source: Nat Commun. 2023 Feb 15;14:853. doi: 10.1038/s41467-023-36606-w (PMC9932158; doi:10.1038/s41467-023-36606-w)
Supplement: Supplementary file 3 — Description of Additional Supplementary Files [file 41467_2023_36606_MOESM3_ESM.pdf]

Description of Additional Supplementary Files

File Name: Supplementary Data 1
Description: Overview and characteristics of the 61 DNMs from the 60 family trios enrolled in the PREGCARE study (with individual details for each step of the three-tier approach: primer locations, regions and results)

File Name: Supplementary Data 2
Description: Targeted deep-sequencing (Deep-NGS) data for the families enrolled in the PREGCARE study

File Name: Supplementary Data 3A
Description: Overview of the phasing strategy for each of the individual PREGCARE families

File Name: Supplementary Data 3B
Description: Description of the informative SNP and read count tables supporting the phasing of DNMs and parent-of-origin determination for individual PREGCARE families

File Name: Supplementary Data 4A
Description: Details of the common single nucleotide polymorphisms (SNPs) used for genotyping the members of family trios and verifying the family relationship (single-molecule Molecular Inversion Probe (smMIP) assay)

File Name: Supplementary Data 4B
Description: Sequence information of the single-molecule Molecular Inversion Probes (smMIP assay)
