# Supplementary material for: Personalized recurrence risk assessment following the birth of a child with a pathogenic de novo mutation
Source: Nat Commun. 2023 Feb 15;14:853. doi: 10.1038/s41467-023-36606-w (PMC9932158; doi:10.1038/s41467-023-36606-w)
Supplement: Supplementary file 5 — Reporting Summary [file 41467_2023_36606_MOESM5_ESM.pdf]

nature portfolio

Corresponding author(s): Anne Goriely

Last updated by author(s): Jan 13, 2023

Reporting Summary

Nature Portfolio wishes to improve the reproducibility of the work that we publish. This form provides structure for consistency and transparency in reporting. For further information on Nature Portfolio policies, see our Editorial Policies and the Editorial Policy Checklist.

Statistics

For all statistical analyses, confirm that the following items are present in the figure legend, table legend, main text, or Methods section.

n/a	Confirmed	
☐	☒	The exact sample size (*n*) for each experimental group/condition, given as a discrete number and unit of measurement
☐	☒	A statement on whether measurements were taken from distinct samples or whether the same sample was measured repeatedly
☐	☒	The statistical test(s) used AND whether they are one- or two-sided<br>*Only common tests should be described solely by name; describe more complex techniques in the Methods section.*
☒	☐	A description of all covariates tested
☒	☐	A description of any assumptions or corrections, such as tests of normality and adjustment for multiple comparisons
☐	☒	A full description of the statistical parameters including central tendency (e.g. means) or other basic estimates (e.g. regression coefficient) AND variation (e.g. standard deviation) or associated estimates of uncertainty (e.g. confidence intervals)
☐	☒	For null hypothesis testing, the test statistic (e.g. *F*, *t*, *r*) with confidence intervals, effect sizes, degrees of freedom and *P* value noted<br>*Give P values as exact values whenever suitable.*
☒	☐	For Bayesian analysis, information on the choice of priors and Markov chain Monte Carlo settings
☒	☐	For hierarchical and complex designs, identification of the appropriate level for tests and full reporting of outcomes
☒	☐	Estimates of effect sizes (e.g. Cohen's *d*, Pearson's *r*), indicating how they were calculated

Our web collection on statistics for biologists contains articles on many of the points above.

Software and code

Policy information about availability of computer code

Data collection	No software was used to collect the data in this study
Data analysis	Commercial and publicly available software used to analyze and visualize data include: amplimap (v0.4.19; incorporates bedtools v2.27.l, BWA v0.7.12 and GATK v4.2.0.0), bcftools (vl.12), Guppy (v4.5.4+66cla77), IGV (v2.11.2), Medaka (vl.3.2), minimap2 (v2.18), NanoStat (vl.5.0), Perl (vS.32.0), Picard Tools (v2.27.2), Python (v3.6.10}, R (v4.2.0), samtools (vl.12). Figures were generated using R v4.2.0 with package ggplot2 v3.3.5, GraphPad Prism v9.2.0, and Adobe Illustrator. Variant calling and haplotyping were performed using standard workflows and programs as described in the methods section of this paper, and available at www.github.com/sjbush/pregcare.

For manuscripts utilizing custom algorithms or software that are central to the research but not yet described in published literature, software must be made available to editors and reviewers. We strongly encourage code deposition in a community repository (e.g. GitHub). See the Nature Portfolio guidelines for submitting code & software for further information.

Data

Policy information about availability of data

All manuscripts must include a data availability statement. This statement should provide the following information, where applicable:

- Accession codes, unique identifiers, or web links for publicly available datasets
- A description of any restrictions on data availability
- For clinical datasets or third party data, please ensure that the statement adheres to our policy

The sequencing data generated during the current study have been deposited in the European Nucleotide Archive under BioProject accession number PRJEB53977 (http://www.ebi.ac.uk/ena/data/view/PRJEB53977).
Additionally, the processed sequencing data relevant to each DNM (Deep-NGS (Supplementary Data 2) and ONT calls (Supplementary Data 3A&B)) are included as Supplementary Material.

Human research participants

Policy information about studies involving human research participants and Sex and Gender in Research.

Reporting on sex and gender	This study aims at characterizing the origin of de novo mutations. Hence, all participant families were recruited as a complete mother-father-child trio. The chromosomal sex of the proband is given in Supplementary Data 1 but is no relevant to the study design or the results. We determined the parental origin of the de novo mutations for each family and in doing so, establish the paternal vs. maternal origin of the DNM. It is well established that there is a sex biased in the origin of de novo mutations, with ~80% of de novo 'one-off' mutations occurring during spermatogenesis. This was confirmed in the present study and discussed in the manuscript, but is not relevant to the participant selection criteria (i.e. both parents are recruited).
Population characteristics	Couples who have had a child with a pathogenic DNM were invited to participate in the PREGCARE study (see below). There are no relevant covariates to the population, only individual couples who were interested to obtain refined recurrence risk for future pregnancies opted to participate after discussion with their clinicians/local clinical team and reading the Participant Information Sheet (PIS). The ages of parents and proband at the time of recruitment into the study are given for each family in Supplementary Data 1 but are not relevant to the study design or the selection criteria.
Recruitment	Couples with one (or multiple) children, stillbirths or terminated pregnancies affected by a likely pathogenic de novo mutation (DNM) and who were potentially interested in personalized transmission risk assessment for future pregnancies, were invited to participate by healthcare professionals during routine clinical genetic consultation. A DNM was defined as a single-nucleotide or small insertion-deletion variant detected in the proband that had been shown to be absent from the parents' DNA on routine diagnostic genetic analysis. The specific method used for original diagnostic of the causative DNM in the proband and for determining the de novo status of the mutation are described in Supplementary Data 1. DNMs in six genes (FGFR2, FGFR3, HRAS, KRAS, PTPN11, RET) known to be associated with the process of 'spermatogonial selection' were excluded from this research study, unless there were multiple affected pregnancies. Couples where the mother was pregnant at the time of sample collection, those who were not both the biological parents of the affected child, or either the biological mother or father did not consent to participate, were also excluded.<br>While a total of 60 mother-father-child trios were recruited into the study, 58 had a single affected child but 2 families (FAM17 and FAM60) had had three pregnancies with the same DNM, indicating that one of the parents must be a gonadal mosaic. To eliminate ascertainment bias, these two families were excluded from the quantitative presentation of the data in the manuscript - but included in the specific analysis of mosaicism. Hence, our primary cohort comprises data from 58 parent-child trios, including one trio with two different pathogenic DNMs (59 DNMs). Recruitment and sample collection took place at 13 of the 17 participating National Health Service (NHS) Trusts in England, UK.
Ethics oversight	The PREGCARE (PREcision Genetic Counselling And REproduction) study protocol was approved by the London - Queen Square Research Ethics Committee under the reference number 17/LO/1025 (IRAS reference: 225264).

Note that full information on the approval of the study protocol must also be provided in the manuscript.

Field-specific reporting

Please select the one below that is the best fit for your research. If you are not sure, read the appropriate sections before making your selection.

☒ Life sciences ☐ Behavioural & social sciences ☐ Ecological, evolutionary & environmental sciences

For a reference copy of the document with all sections, see nature.com/documents/nr-reporting-summary-flat.pdf

Life sciences study design

All studies must disclose on these points even when the disclosure is negative.

Sample size	This is a descriptive study aimed at developing a systematic strategy to stratify de novo mutations (DNMs) into 7 discrete categories and derive personalized recurrence risk in future pregnancies. Based on literature review, the proportion of cases for each of the 7 categories

	defined in Figure 1 were anticipated to range from 2%-71%. Hence we determined that a sample size of ~50 trios would be needed to find representative examples for each one of the categories . In this study, we collected samples from 60 family trios and obtained results for each DNM. Our data showed that, within this clinical cohort, we found DNMs within each one of the 7 different categories presented on Figure 1, confirming that we had obtained a representative sampling of DNM origin.
Data exclusions	Deep-sequencing data were excluded from the analysis, if they did not reach a minimum read depth of ~350x. In these cases, the data was considered void and the sample a 'failure' denoted by NA (Not Available) in Supplementary Data 2 and a X on the associated figures. Furthermore, as described in the text, while a total of 60 couples were recruited into the study, 2 families (FAM17 and FAM60) had three affected pregnancies, indicating that one of the parents must be a gonadal mosaic. To eliminate ascertainment bias, these two families were excluded from the quantitative presentation of the data but included in the specific analysis of mosaicism. Hence, our primary cohort comprises data from 58 parent-child trios, including one trio with two different pathogenic DNMs (59 DNMs).
Replication	This is a descriptive study of a unique cohort comprising family trios with a specific DNM (each family required the development of a custom assay, targeting the unique DNM identified in the proband). The results consist in stratification of risk in one of the 7 categories presented in Figure 1 and Figure 4. No further replication or validation of the data was attempted.
Randomization	This is a descriptive study, randomization is not relevant: all biological samples from a given family were processed (i.e. extracted, amplified, and sequenced) together as a single library.
Blinding	This is a descriptive study, blinding is not relevant.

Reporting for specific materials, systems and methods

We require information from authors about some types of materials, experimental systems and methods used in many studies. Here, indicate whether each material, system or method listed is relevant to your study. If you are not sure if a list item applies to your research, read the appropriate section before selecting a response.

Materials & experimental systems

n/a	Involved in the study
☒	☐ Antibodies
☒	☐ Eukaryotic cell lines
☒	☐ Palaeontology and archaeology
☒	☐ Animals and other organisms
☒	☐ Clinical data
☒	☐ Dual use research of concern

Methods

n/a	Involved in the study
☒	☐ ChIP-seq
☒	☐ Flow cytometry
☒	☐ MRI-based neuroimaging
